# Subclinical Atherosclerosis and Endothelial Dysfunction in Cases of Systemic Lupus Erythematosus: A Hospital-Based Case-Control Study

**DOI:** 10.7759/cureus.67473

**Published:** 2024-08-22

**Authors:** Tapobrata Biswas, Silima Tarenia, Sudipta Bera, Sourav Naiya, Sourya Acharya, Samarth Shukla, Sunil Kumar, Nikhil Pantbalekundri, Anamika G Giri

**Affiliations:** 1 Department of Internal Medicine, Deben Mahata Government Medical College and Hospital, Purulia, IND; 2 Department of Endocrinology, Diabetes, and Metabolism, Nil Ratan Sircar Medical College and Hospital, Kolkata, IND; 3 Department of Pathology, Deben Mahata Government Medical College and Hospital, Purulia, IND; 4 Department of Community Medicine, Deben Mahata Government Medical College and Hospital, Purulia, IND; 5 Department of Medicine, Jawaharlal Nehru Medical College, Datta Meghe Institute of Medical Sciences, Wardha, IND; 6 Department of Pathology, Jawaharlal Nehru Medical College, Datta Meghe Institute of Medical Sciences, Wardha, IND; 7 Department of Internal Medicine, Jawaharlal Nehru Medical College, Datta Meghe Institute of Medical Sciences, Wardha, IND; 8 Department of Internal Medicine, Jawaharlal Nehru Medical College and Acharya Vinoba Bhave Rural Hospital, Wardha, IND

**Keywords:** fmd, carotid intima-media, endothelial dysfunction, atherosclerosis, sle

## Abstract

Background

Women are more likely to be affected with systemic lupus erythematosus (SLE), a chronic multisystem inflammatory autoimmune illness. It is well established that SLE increases the risk of cardiovascular (CV) events. This study aimed to determine the prevalence of endothelial dysfunction and subclinical atherosclerosis in patients with SLE. If these conditions are identified early, suitable preventative measures may be advocated to lessen the burden of future CV events.

Aim

This study aims to calculate the frequency of endothelial dysfunction and subclinical atherosclerosis in SLE patients using the flow-mediated dilatation (FMD) of the brachial artery and carotid intima-media thickness (c-IMT).

Results

There were 50 confirmed cases of SLE. Compared to healthy controls, SLE patients' c-IMT readings were higher, suggesting subclinical atherosclerosis. Thirty-three patients with c-IMT values >0.06 (p<0.00269) out of 50 SLE cases were found to have a high prevalence of subclinical atherosclerosis. Five SLE patients showed FMDs of less than 4.5% (p<0.021) compared to healthy controls, indicating preclinical atherosclerosis with endothelial dysfunction. It was discovered that endothelial dysfunction exhibited a positive linear connection with erythrocyte sedimentation rate (ESR) and C-reactive protein (CRP) when contrasted with traditional inflammatory indicators such as ESR and CRP.

Conclusion

Patients with SLE face a higher risk of CV events and mortality compared to those without the condition. They are also more prone to developing endothelial dysfunction and subclinical atherosclerosis. Detecting these issues early can help in implementing primary and secondary prevention strategies effectively.

## Introduction

Systemic lupus erythematosus (SLE) is a chronic inflammatory autoimmune disease that affects multiple organ systems, primarily impacting young women but also affecting men [1,2]. Individuals with SLE have a significantly higher risk of 10 to 50 times cardiovascular (CV) events due to their predisposition to atherosclerosis [3]. The atherosclerotic process often begins in childhood for those with SLE, leading to chronic inflammation and CV events. Early detection of subclinical atherosclerosis can help apply appropriate preventive treatments [4]. In SLE patients, persistent inflammation is closely linked to atherosclerosis. Additionally, many SLE patients have lupus anticoagulant (LAC) or related antiphospholipid antibodies (APLA), which are prothrombotic and atherogenic, leading to a hypercoagulable state and increased CV risk [5,6].

This study aims to quantify the burden of endothelial dysfunction and subclinical atherosclerosis in SLE patients. Specifically, it seeks to measure carotid intima-media thickness (c-IMT) to estimate the prevalence of subclinical atherosclerosis, assess Flow-mediated dilatation (FMD) of the brachial artery to determine the prevalence of endothelial dysfunction, and correlate these findings with inflammatory biomarkers like C-reactive protein (CRP) and erythrocyte sedimentation rate (ESR).

## Materials and methods

The Institutional Ethics Committee of Calcutta National Medical College and Hospital (CNMCH) approved a case-control study conducted by the Department of General Medicine (CNMC-IEC: 3). The study included 50 adult SLE patients aged 18 to 60, who met the criteria set by the Systemic Lupus International Collaborating Clinics (SLICC) and the American College of Rheumatology (ACR), along with 50 age- and sex-matched healthy controls. Subclinical atherosclerosis was assessed using c-IMT, while endothelial dysfunction was measured by the brachial artery's FMD. The study excluded SLE patients who smoked, drank alcohol, or had diabetes, obesity (BMI >25 kg/m²), hypertension, dyslipidemia, stroke, other peripheral vascular diseases, chronic kidney disease, or any other acute systemic illness or infection. Also excluded were SLE patients on beta-blockers, estrogen, thyroxine, oral contraceptives, lipid-lowering medications, and steroids. Participants underwent thorough medical history collection, anthropometric measurements, and clinical examinations. Blood samples were taken for routine tests, including complete blood count (CBC), urea, creatinine, lipid profile, fasting and post-prandial blood glucose, anti-double-stranded DNA (dsDNA), ESR, and CRP. Ultrasound was used to measure the thickness of the carotid artery intima-media and the brachial artery's FMD.

Flow-mediated dilatation measurement

The internal diameter of the brachial artery was measured using a Doppler Ultrasonogram 7.5 MHZ scanning probe in the B-mode. The mean of three consecutive measurements of the brachial artery internal diameters (2-6 cm above the elbow) was determined at the end-diastole, which was timed by the QRS complex. Following the recording of the brachial artery diameter baseline measurements, forearm ischemia was produced by inflating the cuff to 200 mmHg (or 50 mmHg over systolic blood pressure) for five minutes. After the cuff was deflated, measurements of the artery diameter were made every 45 to 60 seconds. The percentage change in the diameter from baseline to reactive hyperemia was used to express FMD of the brachial artery.

Carotid intima-media thickness measurement

Cases were examined in the supine position. The neck was positioned with a special pillow at a 45° angle. Unaware of the participant's clinical features, one examiner obtained B-mode ultrasound images using a 7 Hz linear array transducer, as advised by the American Society of Echocardiography consensus statement. At this hospital, Aloka Pro sound in B-mode was used to scan both common carotid arteries (CCAs) 1 cm before the carotid bulb along a length of 1 cm at the far wall of both CCAs. The distance between the media-adventitia interface and the leading edge of the lumen-intima interface was used to measure IMT thickness solely at plaque-free segments. After averaging two measurements from each side, the mean c-IMT was determined by averaging the values from both CCAs. The most recent ESH/ESC guidelines for hypertension (2013) state that c-IMT >0.6 mm is considered a sign of silent organ damage. Statistical analysis was done using IBM-SPSS statistical package version 24.

## Results

The mean patient age in the SLE group was 36.4 and 36.8 in healthy volunteers. The disease duration was 5.1+/-1.7 years. The age and sex distribution were similar in the healthy arm. Males had a higher prevalence of subclinical atherosclerosis (Table 1).

**Table 1:** Baseline characteristics of case and control SD: standard deviation; c-IMT: carotid intima-media thickness; FMD: flow-mediated dilatation

Variable	Cases (n=50)	Controls (n=50)	P-value
A. Age (years)			
Mean +/-SD	37+/-11.3	34+/-9.8	p=0.196
B. Gender			
Female	44 (88%)	46 (92%)	p=0.505
Male	6 (12%)	4 (81%)	
C. c-IMT			
0.03-0.06	17 (34%)	32 (64%)	p=0.0026
>0.06	33 (66%)	18 (36%)	
D. FMD			
<4.5	5 (10%)	0	p=0.02178
>4.5	45 (90%)	50 (100%)	

Among the SLE patients, 33 had a c-IMT of more than 0.06 (p<0.027), which indicated that they had subclinical atherosclerosis, whereas 18 healthy controls had abnormal values of c-IMT. Among the SLE patients, five had an FMD of less than 4.5%(p <0.021), which indicated that they had subclinical endothelial dysfunction, whereas none of the healthy controls had an FMD of less than 4.5%. The proportion of abnormal c-IMT in SLE patients above 40 years (76.19%) is higher than the SLE patients below 40 years (58.62%). However, the proportion of abnormal c-IMT in healthy controls also increases with age, with those above 40 years old having a higher proportion (57.14%) compared to controls below 40 years old (Table 2).

**Table 2:** Distribution of c-IMT values as per age in cases and controls c-IMT: carotid intima-media thickness

	Age <40 yrs		Age >40 yrs	
c-IMT	Case	Control	Case	Control
0.03-0.06 cm	12 (41.3%)	26 (72.2%)	5 (23.8%)	6 (42.8%)
>0.06	17 (58.6%)	10 (27.77%)	16 (76.19%)	8 (57.14%)
Total	29	36	21	14

This study shows an increased prevalence of endothelial dysfunction with the rising value of ESR, that is, increased disease activity (Table 3). This study also shows an increased prevalence of FMD with the rising value of CRP (Table 4).

**Table 3:** c-IMT and FMD with rising ESR levels ESR: erythrocyte sedimentation rate; c-IMT: carotid intima-media thickness; FMD: flow-mediated vasodilation

	ESR (first hr) <50	ESR (first hr) 51-70	ESR (first hr) >70	
c-IMT				
c-IMT >0.06 cm	3 (50%)	13 (61.9%)	18 (78.2%)	p=0.306
c-IMT <0.06 cm	3 (50%)	8 (38.1%)	5 (21.8%)	
TOTAL	6	21	23	
FMD				
FMD <4.5%	0 (0%)	3 (15%)	2 (9%)	p=0.922
FMD >4.5%	6 (100%)	17 (85%)	22 (91%)	
TOTAL	6	20	24	

**Table 4:** c-IMT and FMD with rising CRP levels CRP: C-Reactive protein; c-IMT: carotid intima-media thickness; FMD: flow-mediated vasodilation

	CRP (mg/dL) <20	CRP (mg/dL) 21-30	CRP (mg/dL) >31	
c-IMT				
c-IMT <0.06 cm	4 (28.6%)	10 (40%)	3 (27.3%)	p=0.667
c-IMT >0.06 cm	10 (71.4%)	15 (60%)	8 (72.7%)	
TOTAL	14	25	11	
FMD				
FMD <4.5%	0 (0%)	2 (10%)	3 (28%)	p=0.195
FMD >4.5%	19 (100%)	18 (90%)	8 (72%)	
TOTAL	19	20	11	

## Discussion

SLE patients have higher mortality rates and a higher frequency of subclinical atherosclerosis. Autoantibodies, immunological complexes, and changes in lipoprotein metabolism are essential factors in the pathophysiology of elevated atherosclerosis in sickle cell disease. Evidence suggests that all stages of atherosclerosis and accelerated atherogenesis are influenced by chronic inflammation. Fertile women are primarily affected by SLE, which can impact any organ system. This cross-sectional study uses FMD and c-IMT to estimate the prevalence of endothelial dysfunction and subclinical atherosclerosis in SLE patients. Patients with SLE are more likely to experience an acute myocardial infarction [7]. Early detection of subclinical atherosclerosis and endothelial dysfunction can reduce the morbidity and death rate associated with SLE patients.

Thickened c-IMT was more common in SLE patients than in the control group (p=0.0002) in the Katarzyna et al. research [8]. Male SLE patients had a higher incidence of endothelial dysfunction and subclinical atherosclerosis than female patients, although the p-value was not significant. According to our research, the percentage of aberrant c-IMT rises in SLE patients compared to controls in both age groups below and above 40. However, the percentage of abnormal c-IMT in SLE patients beyond 40 (76.19%) is higher than in SLE patients below 40 (58.69%). According to other research, the prevalence of atherosclerotic plaque stays higher in all age groups, especially in younger patients, even when age groups start to diverge. Five SLE patients (p<0.021) had an FMD of less than 4.5%, suggesting preclinical atherosclerosis; in contrast, none of the healthy controls had an FMD of less than 4.5%. According to a study conducted by Sofia Ajeganova et al., patients with SLE who were followed up for 10 years experienced more unfavorable CV events than controls (p=0.022) [9]. Our investigation found a correlation between elevated ESR readings and the incidence of endothelial dysfunction and subclinical atherosclerosis. Endothelium-dependent vasodilatation was reported to be severely reduced by SLE, according to Kiss et al. [10]. In lupus patients, measuring FMD is a helpful way to identify endothelial dysfunction. The decreased ability of the brachial artery to distinguish between individuals with SLE and healthy individuals, as well as between lupus patients with and without atherosclerotic vascular problems, may be helpful. Additionally, a correlation was found between elevated CRP levels and the incidence of endothelial dysfunction and preclinical atherosclerosis. TaPanaFidina discovered a favorable relationship between the illness activity and the high-sensitivity C-Reactive protein (hs-CRP) level. The SLE patients in that study were younger and had higher Hs-CRP levels than the control groups [11]. According to Alarco GS et al., there are variations in high disease activity levels throughout the illness [12]. The mortality and morbidity rates for SLE patients should decrease with the introduction of more advanced anti-inflammatory and immune-suppressive medications [13,14]. The main limitation of the study was that the study population was small. A total of 50 health controls and 50 patients between the age group of 18 to 60 years were included. Therefore, the results of the study may not encourage many other epidemiological studies conducted elsewhere, as this is a hospital-based study and the results may not accurately reflect disease prevalence in the community.

## Conclusions

Patients with SLE have a higher incidence of developing subclinical atherosclerosis and a higher risk of CV events and death compared to those without SLE. Sensitive non-invasive techniques for detecting subclinical atherosclerosis and endothelial dysfunction include c-IMT and flow-mediated vasodilation. Subclinical atherosclerosis and endothelial dysfunction should be assessed in SLE patients in order to allow for the early detection and prevention of potential catastrophic CV events.
